# Associations Between Built Environment Factors and SARS-CoV-2 Infections at the Neighbourhood Level in a Metropolitan Area in Germany

**DOI:** 10.1007/s11524-022-00708-5

**Published:** 2023-01-12

**Authors:** Dennis Schmiege, Timo Haselhoff, Salman Ahmed, Olympia Evdoxia Anastasiou, Susanne Moebus

**Affiliations:** 1 Institute for Urban Public Health (InUPH), University Hospital Essen, University of Duisburg-Essen, 45130 Essen, Germany; 2 Institute of Virology, University Hospital Essen, University of Duisburg-Essen, 45147 Essen, Germany

**Keywords:** COVID-19, Environmental health, Small-scale, Spatial analysis, Urban health

## Abstract

COVID-19-related health outcomes displayed distinct geographical patterns within countries. The transmission of SARS-CoV-2 requires close spatial proximity of people, which can be influenced by the built environment. Only few studies have analysed SARS-CoV-2 infections related to the built environment within urban areas at a high spatial resolution. This study examined the association between built environment factors and SARS-CoV-2 infections in a metropolitan area in Germany. Polymerase chain reaction (PCR)-confirmed SARS-CoV-2 infections of 7866 citizens of Essen between March 2020 and May 2021 were analysed, aggregated at the neighbourhood level. We performed spatial regression analyses to investigate associations between the cumulative number of SARS-CoV-2 infections per 1000 inhabitants (cum. SARS-CoV-2 infections) up to 31.05.2021 and built environment factors. The cum. SARS-CoV-2 infections in neighbourhoods (median: 11.5, IQR: 8.1–16.9) followed a marked socially determined north–south gradient. The effect estimates of the adjusted spatial regression models showed negative associations with urban greenness, i.e. normalized difference vegetation index (NDVI) (adjusted *β* =  − 35.36, 95% CI: − 57.68; − 13.04), rooms per person (− 10.40, − 13.79; − 7.01), living space per person (− 0.51, − 0.66; − 0.36), and residential (− 0.07, 0.16; 0.01) and commercial areas (− 0.15, − 0.25; − 0.05). Residential areas with multi-storey buildings (− 0.03, − 0.12; 0.06) and green space (0.03, − 0.05; 0.11) did not show a substantial association. Our results suggest that the built environment matters for the spread of SARS-CoV-2 infections, such as more spacious apartments or higher levels of urban greenness are associated with lower infection rates at the neighbourhood level. The unequal intra-urban distribution of these factors emphasizes prevailing environmental health inequalities regarding the COVID-19 pandemic.

**Supplementary Information:**

The online version contains supplementary material available at 10.1007/s11524-022-00708-5.

## Introduction

The COVID-19 (coronavirus disease 2019) pandemic has severely affected peoples’ lives globally. COVID-19-related health outcomes, including infections with the pathogen SARS-CoV-2 (severe acute respiratory syndrome coronavirus 2), were not distributed equally between [1, 2] or within countries [3, 4], revealing distinct geographical patterns at different spatial scales. Metropolitan and urban areas were particularly affected [5, 6] due to some defining characteristics, e.g. high population density and high scales and mobility of human activities [7], specifically during the onset of the COVID-19 pandemic. Virus transmission like SARS-CoV-2 is crucially impacted by close spatial proximity of people [8], a distinct feature of metropolitan and urban areas, which in turn could be influenced by the built environment.

The built environment is defined here as human-made spaces, which provide settings for different human activities [9], e.g. residential and commercial areas, green space, and housing conditions. Built environment factors play an essential role in the conceptual link between environment and health [10]. Depending on certain characteristics, e.g. availability, access or quality, and the local context, human-made spaces can harm or promote health via direct and indirect pathways [11], making those important determinants of health and well-being. The built environment can also affect infectious disease transmissions and is therefore a crucial component to consider in the management of the COVID-19 pandemic [12].

Analysing the geographical patterns of COVID-19-related outcomes, the majority of studies focused on compositional factors, i.e. individual-related variables such as age, gender, immigration status, and income, at national [13, 14], sub-national [15, 16], or city level [17, 18]. Only few studies investigated the role of the built environment adopting a small-scale perspective, i.e. examining intra-city differences [19, 20], but none of them in Germany. Such high-resolution analyses are crucial as they can reveal information that would remain hidden at higher aggregated levels and help to tailor interventions to the local context [21].

The objectives of this study were therefore twofold: first, analyse the intra-urban spatial distribution of SARS-CoV-2 infections at a small scale in a metropolitan area in Germany, and second, examine associations between built environment factors and the spatial variation of SARS-CoV-2 infections.

## Methods

Investigating the spatial pattern and associations between SARS-CoV-2 infections and built environment factors, the retrospective analysis was carried out at the population level in the city of Essen, Germany. The reporting of this study follows the STROBE (Strengthening the Reporting of Observational Studies in Epidemiology) statement [22]. The ethics commission of the medical faculty of the University of Duisburg-Essen approved this study (registration number: 21–10242-BO).

### Study Area and Spatial Resolution

The city of Essen, the tenth most densely populated city in Germany [23], is located in the centre of the Ruhr Metropolis, an urban agglomeration of more than 5 million inhabitants in the western part of Germany. The city displays distinct social and ethnic segregation, with a socio-economic north–south gradient [24]. Administratively, Essen comprises nine city districts, which are divided into 50 boroughs. Since the first officially reported COVID-19 case in Essen on 29.02.2020, the city experienced several waves of infection triggered by different virus variants accumulating to more than 200,000 confirmed cases and around 850 deaths (as of December 2022, [25]).

Data in this study was analysed at the highest spatial resolution available, the neighbourhood level (*n* = 426). Developed by a joint working group consisting of city departments and academic institutes, the following criteria were applied to demarcate neighbourhoods [26]: delimitation by joining building blocks; consideration of topographical boundaries, barriers, building and social structure; development of population; no overlap of district borders; and a minimum of 1000 inhabitants.

### Dataset: Overview of Outcome and Explanatory Variables

Between 01.03.2020 and 31.05.2021, the Essen University Hospital (UME) received nasopharyngeal swab samples for polymerase chain reaction (PCR) tests systematically by the local health agency of Essen, covering the first three waves of infections. For the analysis, we used a dataset containing information on 142,418 PCR tests performed during this period. Of those tests, 67,129 tests (47.1%) were carried out on residents of Essen. After removing double positive PCR tests from the same person within the last 60 days, 7866 individual cases of PCR-confirmed SARS-CoV-2 infections remained. This indicates that around 31% of all positive PCR tests in Essen during the study period (*n* = 25,667) were carried out at UME.

Testing the validity of the UME dataset, we compared the calculated 7-day incidence to the officially reported incidence for the city of Essen by the federal Robert Koch Institute [27], showing a high similarity (Pearson correlation, *R*^2^ = 0.91, see also Fig. A in supplementary material). Regarding the age and sex distribution, the study population in the UME dataset exhibited a slightly higher percentage of young (below age 14) and elderly (above age 60) individuals, as well as a lower percentage of males and higher percentage of females.

Due to data protection, the number of individual PCR tests was aggregated into neighbourhoods (*n* = 426) for analysis. As the residential address was available for each case (*n* = 7,866), all individual PCR tests could be assigned to their respective neighbourhoods. The outcome is the cumulative number of people infected with SARS-CoV-2 (PCR-confirmed) per 1000 inhabitants (inh.) in neighbourhoods up to 31.05.2021. This number was calculated by dividing the number of people infected with SARS-CoV-2 (PCR-confirmed) by the number of all residents in a neighbourhood, the latter being provided by the City of Essen, based on the residents’ registration office.

For the statistical analysis, several demographic (i.e. population density, number of people in different age groups), socio-economic (i.e. number of people without German nationality, welfare recipients or unemployed persons), and built environment variables were available at the neighbourhood level (for a complete list of all available variables, see Table A in supplementary material). The Office for Statistics, Urban Research and Elections of the city of Essen prepared this data in collaboration with other institutions specifically for the neighbourhood level, making it more recent than census data. As previous studies investigated the role of demographic and socio-economic factors in Germany [28, 29], we focus here on the built environment. Table 1 depicts the built environment factors used individually as expositions in the analysis.

**Table 1:** Built environment factors at the neighbourhood level with their year, description, and data source

Built environment factors	Year	Description	Data source
Residential area (%)	2019	Proportion of the area of all residential buildings	Ruhr Regional Association
Residential area with multi-storey buildings (%)	2019	Proportion of the area of all residential buildings higher than three floors	Ruhr Regional Association
Commercial area (%)	2019	Proportion of the area of all commercial buildings	Ruhr Regional Association
Green space (%)	2019	Proportion of the area of public and private green spaces, including parks, open spaces near houses, meadows, pastures and forests	Ruhr Regional Association
Normalized difference vegetation index (NDVI)	2020 (May)	Proportion of the near-infrared and visible parts of the sun radiation reflected by vegetation	USGS Earth Explorer, Landsat 8
Rooms per person	2020	Ratio of the number of rooms and the number of persons in an area	Office for Statistics, Urban Research and Elections, City of Essen
Living space (m^2^) per person	2020	Ratio of the living space in square meters and the number of persons in an area	Office for Statistics, Urban Research and Elections, City of Essen

Residential and commercial areas, as well as green space, were calculated as the proportion of the respective land use type to the whole area in a neighbourhood. The NDVI was calculated for each neighbourhood based on data from the OLI sensor (Operational Land Imager) of the Landsat 8 satellite (May 2020, no visible cloud cover) obtained from United States Geological Survey website at a 30 m^2^ resolution, using ArcGIS Pro (version 2.9.0). It is a commonly used indicator, ranging between − 1 and + 1 [30], to measure the mass of green leaf vegetation of an area, i.e. indicating its greenness [31], as healthy dense vegetation absorbs more visible radiation and reflects larger portions of near-infrared radiation as opposed to sparse vegetation. Rooms and living space per person were derived by dividing the number of rooms and the living space of all residential buildings by the number of inhabitants in each neighbourhood.

### Data Analysis: Map Production, Descriptive and Inferential Statistics

All statistical analyses were implemented using R (version 4.2.1). For the descriptive analysis, the median and interquartile ranges (IQR) were used because most variables exhibited right skewed distributions (see Fig. B in supplementary material). Investigating spatial patterns, the outcome and explanatory variables were mapped in ArcGIS Pro (version 2.9.0). As expected due to the spatial nature of the data, the thematic maps suggested spatial autocorrelation for several variables, which were confirmed by calculating global Moran’s *I* (see Table B in supplementary material). In preparation for that, we computed a spatial weighting matrix using the longitude and latitude of the centroids of each neighbourhood. Due to the high heterogeneity in the size of the neighbourhoods, the first-order queen contiguity matrix was used, which defines areas as neighbours if they share a border with the respective neighbourhood [32]. We used basic binary coding to assign spatial weights.

To assess the influence of the built environment factors on the cumulative number of SARS-CoV-2 infections at a small scale, we implemented simple and multiple linear and spatial regression analyses (for a detailed description of the methods applied in the context of COVID-19, see, e.g. [1, 2]). In order to select variables for the multivariate analysis, minimal sufficient adjustment sets for estimating direct effects of the expositions on the outcome were derived based on the available data using directed acyclic graphs (DAGs) [33] (see Fig. C in supplementary material).

Ordinary least squares (OLS) regression was used to compute the effect of each variable individually. OLS regression models rely on the assumptions of homogeneity and spatial non-variability of the residual errors [34]. When spatial autocorrelation is present, the effect estimators of the OLS regression may be biased and inconsistent [35]. We tested the residuals of each OLS regression model for spatial autocorrelation by computing global Moran’s *I*. In addition, we applied geographically weighted regression (GWR) to explore how the associations between the variables may vary over space [36]. GWR implements a regression model for each neighbourhood, taking into account a specific number of the surrounding neighbours [37]. The GWR models were specified by using an adaptive kernel with varying bandwidth (Gaussian kernel function), whereby the optimal bandwidth, i.e. the proportion between 0 and 1 of observations to include in the k-nearest neighbours weighting scheme, was determined by drop-1 cross-validation.

To account for spatial interactions, we used spatial autoregressive models, namely, mixed spatial lag models (SLM). The SLMs incorporate spatial dependency between the parameters by adding spatially lagged dependent and explanatory variables based on a previously defined spatial weights matrix [38]. Each exposition was adjusted individually for confounding based on the minimal sufficient adjustment sets derived from the DAGs to estimate their direct effects (see Fig. C in supplementary material). We checked for multi-collinearity in the models by using the variance inflation factor (VIF) [39], removing variables with VIF values greater than five (see Table E in supplementary material). For each effect estimator, corresponding 95% confidence intervals (CI) and Nagelkerke pseudo-*R*^2^, a goodness-of-fit measure indicating how well the model explains the data, are provided.

## Results

### Spatial Pattern of PCR-Confirmed SARS-CoV-2 Infections and Built Environment Characteristics of Neighbourhoods

**Fig. 1:**
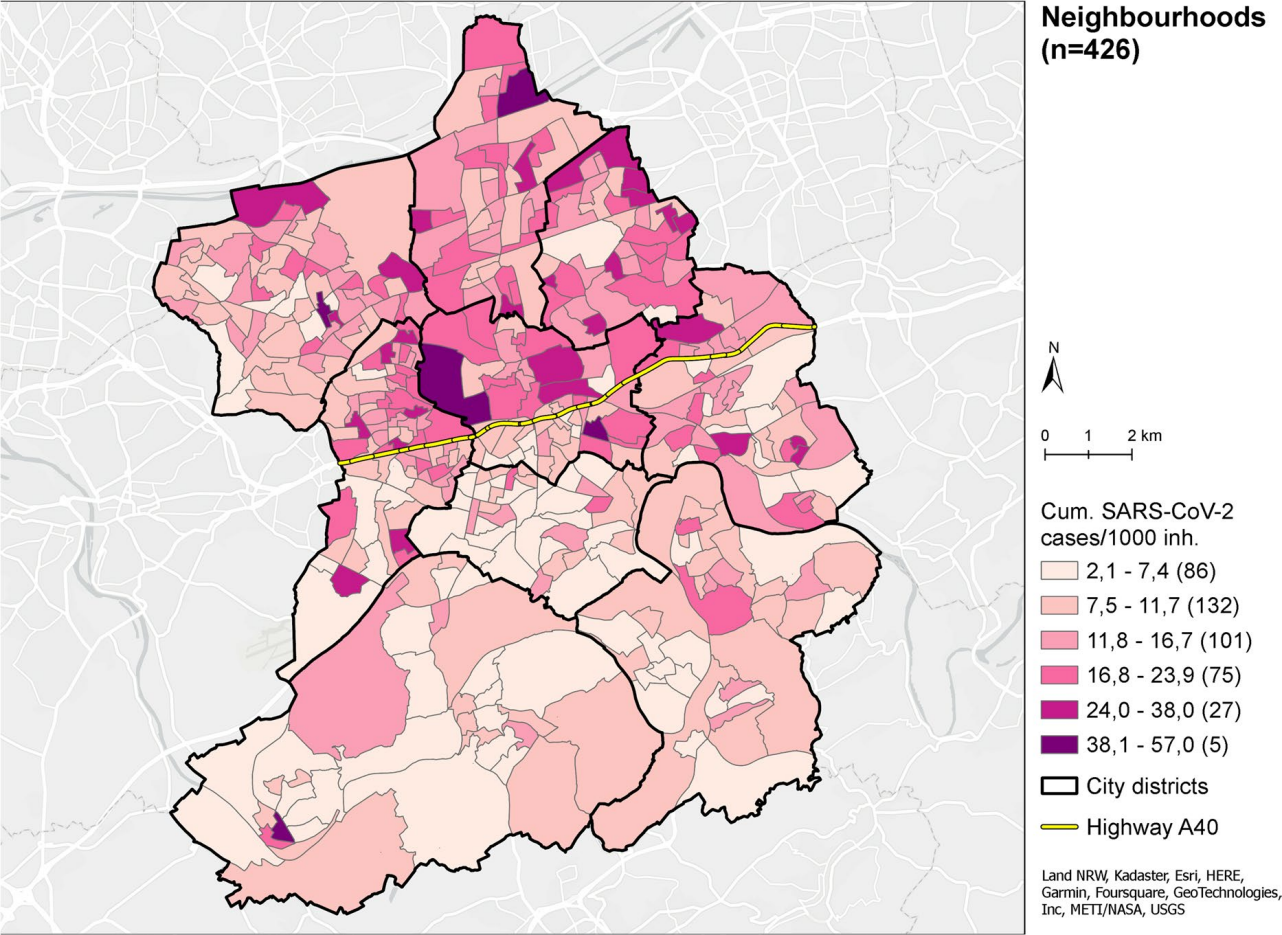
Cumulative SARS-Cov-2 cases (PCR-confirmed) per 1000 inhabitants in Essen at neighbourhood level up to 31.05.2021 (data sources: UME data and city of Essen)

At the end of the study period (31.05.2021), neighbourhoods displayed, on average (median), 11.5 SARS-CoV-2 infections/1000 inh (IQR: 8.1–16.9, min: 2.1, max: 57.0). The cumulative numbers of SARS-CoV-2 infections were not distributed equally across the city of Essen (see Fig. 1, global Moran’s *I* = 0.17, *p* < 0.001), exhibiting a north–south gradient whereby northern neighbourhoods (relative to the highway A40) tend to display higher relative case counts.

Descriptive statistics of the built environment factors across all neighbourhoods and for three groups based on the distribution of the cumulative number of SARS-CoV-2 infections revealed further potential associations (see Table 2).

**Table 2:** Built environment characteristics across all neighbourhoods (median and IQR) and stratified into three groups based on the cumulative number of SARS-CoV-2 infections up to 31.05.2021 in Essen

		Cumulative SARS-CoV-2 infections/1000 inh		
	All (n = 426)	< 9 (*n* = 138)	≥ 9– < 14.5 (*n* = 143)	≥ 14.5 (*n* = 145)
Built environment factors	Median [Q1–Q3]			
Residential area (%)	34.4 [20.9–46.4]	32.4 [18.2–45.1]	37.7 [23.7–49.4]	33.4 [22.1–45.9]
Residential area with multi-storey buildings (%)	11.7 [5.2–23.0]	9.3 [2.8–15.5]	13.6 [5.9–24.6]	13.0 [6.6–26.9]
Commercial area (%)	4.3 [1.2–12.0]	2.9 [1.0–9.2]	4.7 [1.2–11.9]	6.0 [1.6–14.1]
Green space (%)	19.7 [12.8–28.2]	22.2 [15.3–32.8]	19.4 [10.6–28.7]	18.6 [11.0–24.6]
Normalized difference vegetation index (NDVI)	0.29 [0.24–0.34]	0.31 [0.26–0.37]	0.28 [0.24–0.33]	0.27 [0.23–0.31]
Rooms per person	2.1 [1.8–2.3]	2.3 [2.0–2.4]	2.1 [1.9–2.3]	1.9 [1.6–2.1]
Living space (m^2^) per person	41.4 [36.6–47.6]	47.3 [41.7–52.5]	42.0 [38.1–47.4]	36.9 [32.4–40.9]

Differences regarding built environment factors in neighbourhoods occur mainly between the group with the lowest cumulative cases (< 9/1000 inh.) compared to the other two groups. Neighbourhoods in the lowest group tend to have lower proportions of residential and commercial areas, residential areas with multi-storey buildings, and a higher proportion of green space, as well as a higher NDVI and number of rooms and living space per person.

### Spatial Regression Analyses of Built Environment Factors

As the input variables and residuals in all OLS regression models (except for rooms and living space per person) showed spatial autocorrelation (see Tables B and C in supplementary material), mixed spatial lag models were employed to estimate the association between each built environment factor and the cumulative SARS-CoV-2 infections/1000 inh. at neighbourhood level. The results of the geographically weighted regression models (see Table D in supplementary material) confirmed this approach, as commercial and residential areas, residential area with multi-storey buildings, green space, and NDVI presented spatially varying effects; only rooms and living space per person demonstrated a constant negative effect across space.

The minimal sufficient adjustment differed slightly for the expositions (see Fig. C in supplementary material). In each spatial regression model, explanatory variables were removed due to multi-collinearity based on the VIF (see Table E in supplementary material). Table 3 displays the results of the spatial regression analyses.

**Table 3:** Associations of built environment factors and cumulative SARS-CoV-2 infections in neighbourhoods in Essen (*n* = 426) estimated via crude and adjusted mixed spatial lag regression models (SLM) with 95% confidence intervals (CI) and Nagelkerke pseudo-*R*^2^

	*β*	95% CI	Pseudo-*R*^2^
Crude			
Residential area (%)	− 0.03	[− 0.08, 0.03]	0.06
Residential area with multi-storey buildings (%)	0.04	[− 0.03, 0.12]	0.07
Commercial area (%)	− 0.02	[− 0.10, 0.06]	0.06
Green space (%)	− 0.03	[− 0.10, 0.04]	0.07
Normalized difference vegetation index (NDVI)	− 11.51	[− 25.76, 2.73]	0.08
Rooms per person	− 11.52	[− 14.15, − 8.89]	0.26
Living space (m^2^) per person	− 0.53	[− 0.64, − 0.41]	0.27
Adjusted models			
Residential area (%)^1^	− 0.07	[− 0.16, 0.01]	0.24
Residential area with multi-storey buildings (%)^2^	− 0.03	[− 0.12, 0.06]	0.24
Commercial area (%)^3^	− 0.15	[− 0.25, − 0.05]	0.24
Green space (%)^4^	0.03	[− 0.05, 0.11]	0.23
NDVI^5^	− 35.36	[− 57.68, − 13.04]	0.23
Rooms per person^6^	− 10.40	[− 13.79, − 7.01]	0.27
Living space (m^2^) per person^6^	− 0.51	[− 0.66, − 0.36]	0.28

SLMs adjusted for: ^1^commercial area, green space, NDVI, population density, residential area with multi-storey buildings, and welfare recipients (as a proxy for the contextual socio-economic status (cont. SES)); ^2^commercial area, green space, NDVI, residential area, and welfare recipients (cont. SES); ^3^green space, NDVI, population density, residential area, residential area with multi-storey buildings, and welfare recipients (cont. SES); ^4^commercial area, NDVI, population density, residential area, residential area with multi-storey buildings, and welfare recipients (cont. SES); ^5^commercial area, green space, residential area, residential area with multi-storey buildings, and welfare recipients (cont. SES); ^6^population density and welfare recipients (cont. SES)

Residential and commercial areas, NDVI, rooms, and living space per person were negatively associated with the cumulative SARS-CoV-2 infections/1000 inh. at the neighbourhood level, whereas residential area with multi-storey buildings and green space did not show any substantial association. After adjustment, a 10%-point increase in residential or commercial areas was linked to 0.7 and 1.5 fewer cases/1000 inh., respectively. A 0.1 increase in NDVI was associated with a decrease of 3.5 cases/1000 inh., and having an additional room or adding one square meter per person at an aggregated level reduces the cumulative cases by 10.4/1000 inh. and 0.5/1000 inh., respectively.

### The Role of Demographic and Socio-Economic Determinants

Although the focus of this study is on the built environment, the role of demographic and socio-economic determinants should not go unmentioned. Several neighbourhood demographic and socio-economic variables were associated with the cumulative number of SARS-CoV-2 infections (see Tables F, G, and H in supplementary material). For instance, the number of children, as well as the number of persons without German nationality, welfare recipients and unemployed persons all demonstrated positive associations with SARS-CoV-2 infections at the neighbourhood scale.

## Discussion

This study demonstrated the associations between built environment factors, i.e. residential and commercial areas, NDVI and housing space, and the cumulative number of SARS-CoV-2 infections at the neighbourhood level in a metropolitan area in Germany. It thereby complements existing studies on the determinants of COVID-19 cases in Germany [28, 29] that did not examine the role of the built environment and offers in addition a more in-depth perspective by using a high spatial resolution.

The observed north–south gradient of SARS-CoV-2 infections in the city of Essen follows the typical socio-spatial structure of cities in the Ruhr Metropolis. Socially disadvantaged neighbourhoods in the north of Essen historically evolved as a result of industrialization and deindustrialization, as they were traditionally built for coal mining and industrial workers [40]. After the end of coal mining, segregation was further reinforced in these neighbourhoods through, for instance, social housing policies so that the characteristic pattern still widely persists [41]. Although cities in the Ruhr Metropolis, including the city of Essen, share a similar socio-economic structure, transferring and generalizing findings from this study to other national and international cities require further validation.

Built-up areas can influence human activity to varying degrees [42], but the influence may vary depending on the specific land use type. Commercial and residential areas showed negative associations with cumulative SARS-CoV-2 infections, which is in contrast to other studies that linked superstores [15], as well as restaurants and public markets [43] to a higher COVID-19 prevalence. Neither land use type could be further specified in our study, implying that effects from different commercial, e.g. supermarkets or office spaces [20, 44], or residential types, single-family houses or high-rise buildings [19], could be masked. Moreover, the negative associations may indicate the influence of spatial mobility, whereas an infection remains possible in such settings, and the resulting PCR-test would be allocated to the home address, which may be located in a different neighbourhood.

### Associations Between Urban Green Space and Greenness and SARS-CoV-2 Infections

Internationally, multiple studies from different countries examined the influence of neighbourhood greenness (i.e. NDVI) or green space on COVID-19 cases and mortality. Urban green spaces provide opportunities for outdoor physical activity [45] and social interaction [46], which are in turn associated with a range of positive health and well-being outcomes [47, 48]. Emerging evidence from countries in the Global North has indicated that people spent more time in green spaces during the COVID-19 pandemic [49, 50]. Urban green spaces as “safe spaces for socializing, physical activity and recreation” [51] may have played an important role in maintaining or improving physical and mental health during the pandemic because they enabled compliance with physical distancing rules while engaging in such activities [52], lowering the infection transmission risk.

Our analysis revealed a negative association between NDVI and SARS-CoV-2 infections after adjusting for potential confounding, indicating that greener neighbourhoods tended to have fewer cumulative cases. Findings in previous studies are in line with these results. Regardless of how urban green space was measured, several studies found an inverse association between COVID-19 cases and urban vegetation [51, 52], (natural) greenness (USA, South Korea) [53] or green streets (USA) [54].

Although neighbourhood greenness was associated with SARS-CoV-2 infections in our study, the proportion of green space showed little to no association. Spotswood and colleagues made a similar observation [51]. Disentangling the variable “green space” into its individual components revealed a positive association between parks and SARS-CoV-2 infections, whereas open spaces near houses and forests were negatively associated, with meadows and pastures displaying no clear association (see Table I in supplementary material). This indicates the importance of considering not just larger scale green spaces, such as parks, but also smaller area interventions to create a greener neighbourhood, e.g. planting tree rows along streets or open spaces. Such small-scale greening efforts in neighbourhoods may prove as an effective addition to constructing large green spaces in densely built urban areas, where free space is usually scarce.

Using the NDVI to assess neighbourhood greenness has certain limitations. A higher NDVI indicates healthy dense vegetation but provides otherwise no insights about its quality regarding availability, accessibility, affordability, or appropriateness. In addition, it is also not feasible to identify the type of vegetation, e.g. lawn, shrubs, and trees, at the neighbourhood level. Future studies could focus on disentangling the underlying mechanisms of how urban neighbourhood greenness affects infectious disease outcomes, as our results indicate that it is not necessarily through the provision of larger urban green spaces, such as parks. Regarding the relatively wide confidence intervals for NDVI, it should be noted that small-scale analyses may result in small sample sizes, which in turn cause higher variance and thus wider confidence intervals. The resulting lower precision and greater uncertainty in the results therefore call for studies with larger sample sizes, which may underline our findings.

### Associations Between Crowded Housing Conditions and SARS-CoV-2 Infections

Crowded housing conditions are associated with a range of adverse health and well-being outcomes, including an increased risk of acquiring respiratory diseases [55]. Whether housing conditions are considered “crowded” depends on different factors [56], but it is often measured by relating available rooms or floor area to the number of occupants [55]. Living in crowded housing conditions during the COVID-19 pandemic made it more difficult (if not impossible) to maintain physical distancing or practice self-isolation in case of an infection, and exposed people living in such conditions more frequently to the virus [57, 58].

Both proxies for housing conditions analysed in this study, i.e. ratio of rooms and living space per person, were negatively associated (after adjusting for potential confounding) with SARS-CoV-2 infections, suggesting that neighbourhoods with more housing space tended to have fewer cumulative cases. Studies in Brazil [59], France [4], and the USA [60] also found positive associations between crowded housing conditions and COVID-19 cases. In addition, overcrowding was linked to a higher COVID-19 mortality [61]. These findings underline the necessity to improve crowded housing conditions, particularly in the context of infectious diseases. Reducing the adverse impacts of crowded housing conditions, potential interventions include extending or refurbishing existing houses, subsidizing social or public housing and building affordable rental housing [55]. As these interventions are rather long term, short-term solutions for the COVID-19 pandemic could entail, for instance, setting up an emergency housing system for people living in crowded housing conditions [62]. Moreover, this provides another example of how socio-economic inequalities amplify negative health effects through the built environment.

One drawback of our ecological analysis of crowded housing conditions is that due to a lack of data, we could not control for other potential risk factors of infection, which may be associated with living in such conditions, like reliance on public transport or occupation in low-paid or high-risk jobs. Along the same lines, our data did not allow to account for spatial mobility. As the home address of individuals was aggregated into neighbourhoods, it is unclear where the virus transmission and infection took place. Neighbourhoods with a high number of cumulative cases, therefore, do not necessarily equal areas with a high infection dynamic. In addition, dividing the number of rooms or the square meters by the number of inhabitants per neighbourhood does not provide any information about small-scale differences within neighbourhoods or the actual housing situation.

### Demographic and Socio-Economic Determinants of COVID-19 Cases in Germany

The role of demographic and socio-economic determinants of COVID-19 cases was investigated at different spatial scales in Germany. At the county level, Ehlert (2021) found positive associations between COVID-19 cases and average age and population density, negative associations with younger children in day care, while unemployment was not significant [28]. Our results contrast these findings, which underlines the necessity for small-scale analyses as such information can be masked at lower spatial resolutions, e.g. the county level. Particularly in the context of a pandemic, data availability and preparation are needed at a high spatial scale to ensure adequate management of such a health emergency.

Straßburger and Mewes (2022) examined the relationship between district socio-economic status score and SARS-CoV-2 infections at the city-district level in Duisburg, a city only a few kilometres west of Essen. Their results suggest that socially disadvantaged districts were particularly affected in the second wave of infection [29]. This is in line with our results, as two of the score components, i.e. welfare recipients and the number of people without German nationality, were positively associated with the cumulative number of SARS-CoV-2 infections/1000 inh. As none of the studies above examined the role of the built environment, our study contributes an important perspective by filling this research gap.

## Conclusion

Our results suggest that built environment factors, particularly housing space and neighbourhood greenness but also residential and commercial areas, matter for the spread of SARS-CoV-2 infections in a metropolitan area in Germany, underlining the relevance and importance to consider the built environment in future analyses. More spacious apartments or higher levels of urban greenness are associated with lower infection rates at the neighbourhood level.

The unequal intra-urban distribution of these factors emphasizes prevailing environmental health inequalities regarding the COVID-19 pandemic in a metropolitan area in Germany. Understanding such small-scale spatial patterns of disease could help local decision-makers to timely employ effective and targeted measures to the most affected neighbourhoods.

## Supplementary Information

Below is the link to the electronic supplementary material.

Supplementary file1 (PDF 675 kb)

## Data Availability

The data supporting the findings of this study are available within the article and its supplementary information file.
