# Supplementary material for: Associations Between Built Environment Factors and SARS-CoV-2 Infections at the Neighbourhood Level in a Metropolitan Area in Germany
Source: J Urban Health. 2023 Jan 12;100(1):40–50. doi: 10.1007/s11524-022-00708-5 (PMC9836336; doi:10.1007/s11524-022-00708-5)
Supplement: Supplementary file 1 — Supplementary file1 (PDF 675 kb) [file 11524_2022_708_MOESM1_ESM.pdf]

Associations between built environment factors and SARS-CoV-2 infections at the neighbourhood-level in a metropolitan area in Germany

Supplementary material

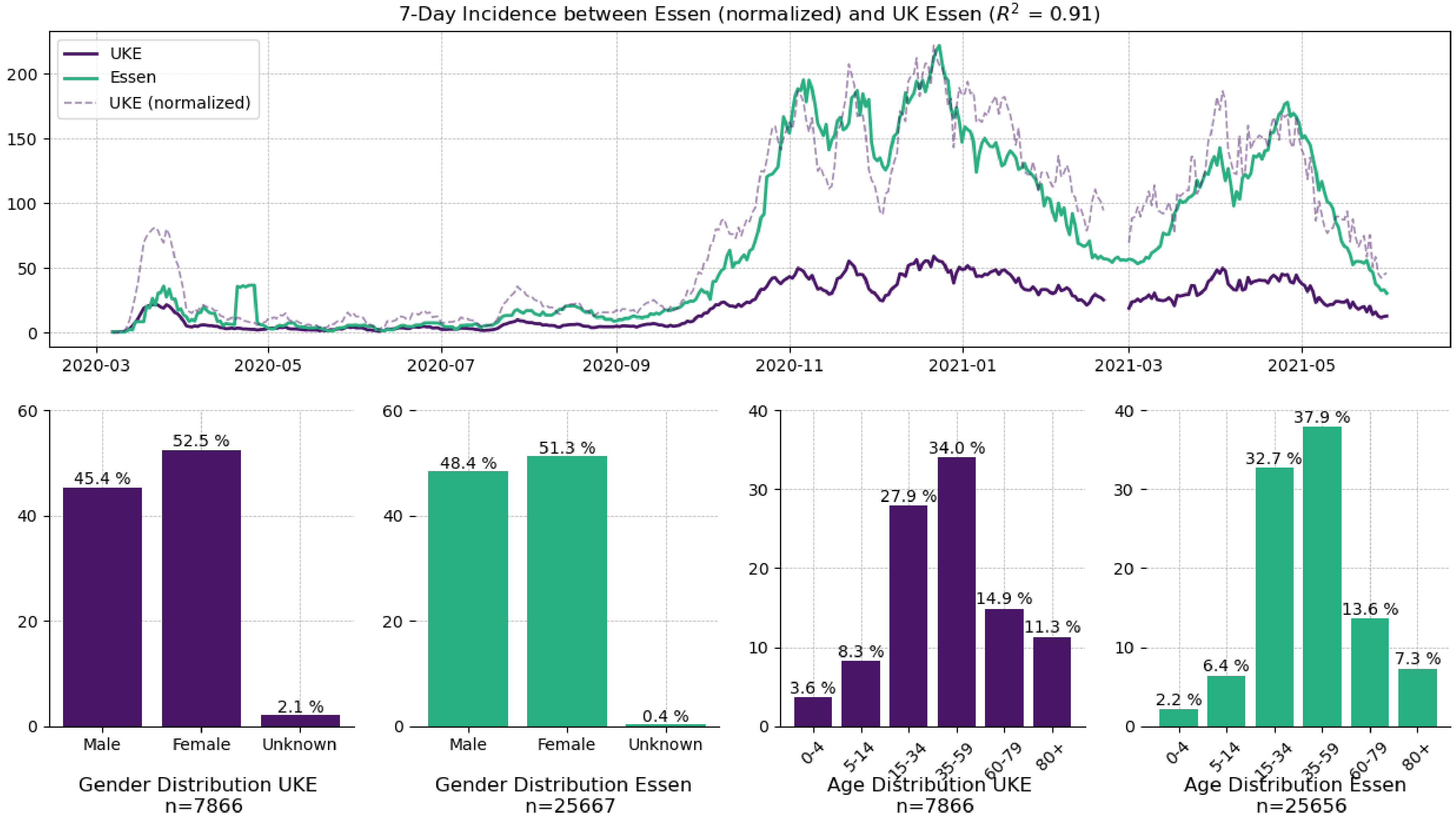

Figure A. Comparison of UME dataset to officially reported data for the city of Essen by the Robert Koch Institute

Table A. List of all demographic, socio-economic and built environment variables at the neighbourhood level with their year, description and data source

	Year	Description	Data source
Demographic			
Number of children below age 6/1000 inh.	2019	Sum of children below age 3 and between age 3 and 5 normalized to neighbourhood population	Office for Statistics, Urban Research and Elections, City of Essen
Number of children and adolescents between age 6 and 19/1000 inh.	2019	Sum of children between age 6 and 10, 10 and 14, 15 and 17, 18 and 19 normalized to neighbourhood population	Office for Statistics, Urban Research and Elections, City of Essen
Number of elderly above age 70/1000 inh.	2019	Sum of persons between age 70 and 74, 75 and 79, and above 80 normalized to neighbourhood population	Office for Statistics, Urban Research and Elections, City of Essen
Average age	2019	Average age of all inhabitants in a neighbourhood	Office for Statistics, Urban Research and Elections, City of Essen
Population density (inh./km²)	2020	Number of inhabitants divided by the area of a neighbourhood	Office for Statistics, Urban Research and Elections, City of Essen
Proportion of households with children (%)	2020	Number of households with children divided by the number of all households in a neighbourhood	Office for Statistics, Urban Research and Elections, City of Essen
Average household size	2019	Number of inhabitants divided by the number of households in a neighbourhood	Office for Statistics, Urban Research and Elections, City of Essen
Socio-economic			
Number of persons without German nationality/1000 inh.	2019	Number of persons without German nationality normalized to neighbourhood population	Office for Statistics, Urban Research and Elections, City of Essen
Number of welfare recipients/1000 inh.	2019	Number of welfare recipients normalized to neighbourhood population	Office for Statistics, Urban Research and Elections, City of Essen
Number of unemployed persons/1000 inh.	2019	Number of unemployed persons normalized to neighbourhood population	Office for Statistics, Urban Research and Elections, City of Essen
Built environment			
Residential area (%)	2019	Proportion of the area of all residential buildings	Ruhr Regional Association
Residential area with multi-storey buildings (%)	2019	Proportion of the area of all residential buildings higher than three floors	Ruhr Regional Association
Commercial area (%)	2019	Proportion of the area of all commercial buildings	Ruhr Regional Association
Green space (%)	2019	Proportion of the area of public and private green spaces, including parks, open spaces near houses, meadows, pastures and forests	Ruhr Regional Association
Normalized Difference Vegetation Index (NDVI)	2020 (May)	Proportion of the near-infrared and visible parts of the sun radiation reflected by vegetation	USGS Earth Explorer, Landsat 8
Rooms per person	2020	Ratio of the number of rooms and the number of persons in an area	Office for Statistics, Urban Research and Elections, City of Essen
Living space (m²) per person	2020	Ratio of the living space in square meters and the number of persons in an area	Office for Statistics, Urban Research and Elections, City of Essen

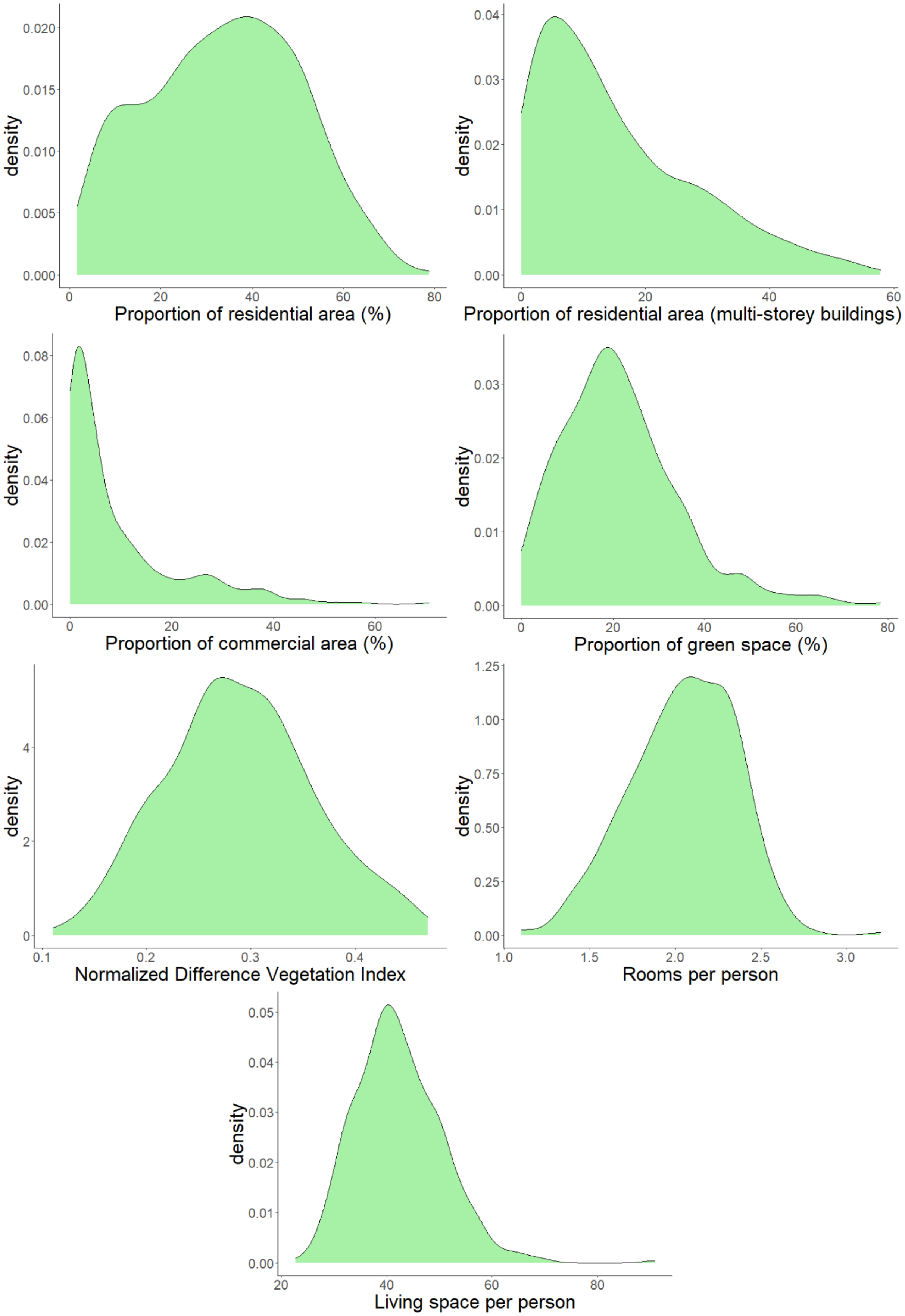

Figure B. Density plots of built environment factors used in the statistical analysis

Table B. Global Moran's I of all built environment factors and cumulative SARS-CoV-2 infections at neighbourhood level (n=426)

	Moran's I statistic	***p-value***
Small-scale built environment		
Residential area (%)	0.32	<.001
Residential area with multi-storey buildings (%)	0.37	<.001
Commercial area (%)	0.42	<.001
Green space (%)	0.44	<.001
Normalized Difference Vegetation Index (NDVI)	0.57	<.001
Rooms per person	0.47	<.001
Living space per person	0.65	<.001
Outcome		
Cumulative SARS-CoV-2 infections/1000 inh.	0.17	<.001

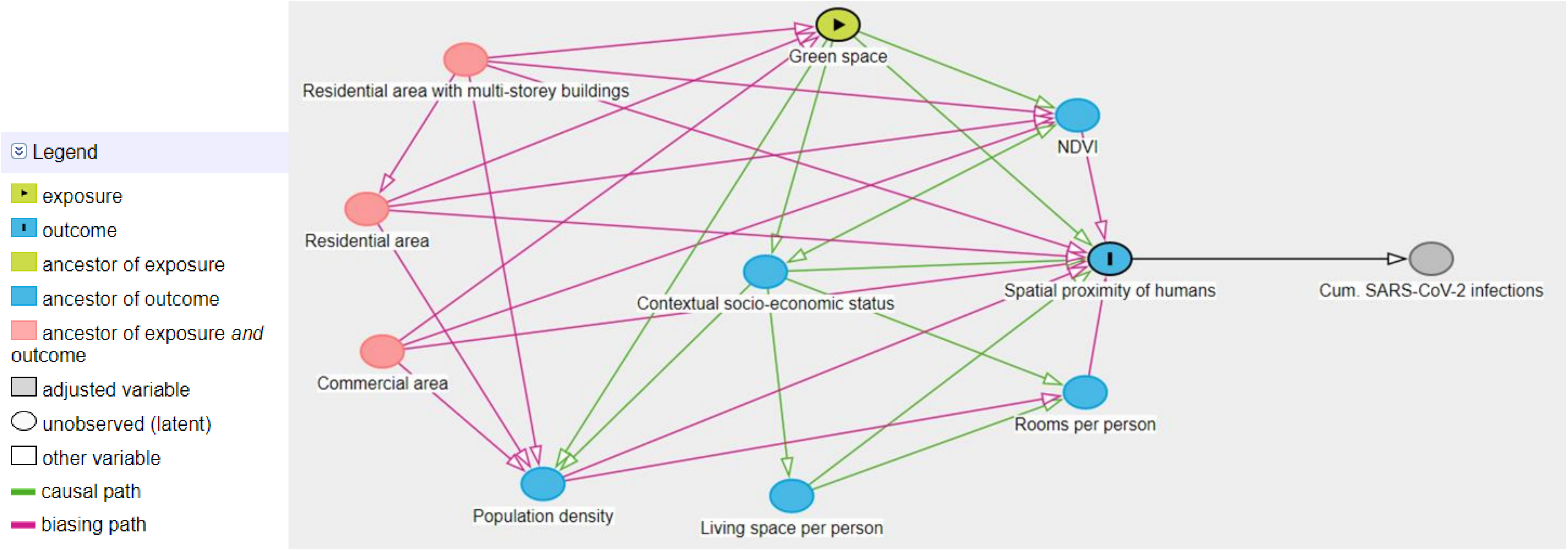

Note: Arrows between boxes were conceptualized as "X influences the probability of the occurrence of Y".

Figure C. Directed acyclic graph (DAG) to derive the minimal sufficient adjustment sets for the expositions in the multivariate analysis, using the example of green space (drawn in http://www.dagitty.net/, developed by Textor et al. (2016)[1])

[1] Textor, J., van der Zander, B., Gilthorpe, M. S., Liśkiewicz, M., Ellison, G. TH (2016): Robust causal inference using directed acyclic graphs: the R package 'dagitty'. International Journal of Epidemiology, Volume 45, Issue 6, December 2016, Pages 1887–1894, https://doi.org/10.1093/ije/dyw341

Table C. Crude effects of built environment factors on cumulative SARS-CoV-2 infections in neighbourhoods in Essen (n=426) estimated via ordinary least squares (OLS) regression models and Moran's I of the regression residuals

	β	95% CI	Std. Err.	Adj. R^2	Moran's I (p-value)
Residential area (%)	-0.02	-0.07;0.02	0.02	-0.0004	0.17 (<.001)
Residential area with multi-storey buildings (%)	0.07	0.01;0.12	0.03	0.009	0.16 (<.001)
Commercial area (%)	0.02	-0.04;0.09	0.03	-0.001	0.17 (<.001)
Green space (%)	-0.08	-0.14;-0.03)	0.03	0.02	0.14 (<.001)
Normalized Difference Vegetation Index (NDVI)	-22.27	-32.51;-12.03)	5.21	0.04	0.13 (<.001)
Rooms per person	-12.73	-14.79;-10.66)	2.18	0.26	0.02 (0.25)
Living space per person	-0.48	-0.56;-0.41)	0.04	0.27	0.02 (0.16)

Table D. Crude associations between built environment factors and cumulative SARS-CoV-2 infections in neighbourhoods in Essen (n=426) estimated via geographically weighted regression (GWR) models

	Min	Q1	Median	Q3	Max	Adaptive quantile (no. of data points)	Quasi-global R^2
Residential area (%)	-0.26	-0.05	-0.02	0.03	0.09	0.04 (18)	0.24
Residential area with multi-storey buildings (%)	-0.18	-0.007	0.05	0.09	0.15	0.06 (27)	0.21
Commercial area (%)	-0.12	-0.05	0.01	0.05	0.22	0.07 (29)	0.18
Green space (%)	-0.21	-0.07	-0.03	-0.007	0.10	0.06 (26)	0.19
Normalized Difference Vegetation Index (NDVI)	-48.57	-24.47	-13.68	-1.47	6.23	0.07 (28)	0.20
Rooms per person	-12.91	-12.71	-12.57	-12.50	-12.36	0.44 (189)	0.26
Living space per person	-0.60	-0.55	-0.52	-0.48	-0.42	0.23 (100)	0.28

Table E. Overview of minimal sufficient adjustment sets based on the available data for the spatial regression models of each exposition derived the DAGs (highlighted in **red** are variables, which were removed during the adjustment process due to a variance inflation factor (VIF) >5)

**Exposition //** *Variables*	*Residential area (%)*	*Residential area with multi-storey buildings (%)*	*Commercial area (%)*	*Green space (%)*	*NDVI*	*Rooms per person*	*Living space (m²) per person*	*Population density*	*Welfare recipients (contextual socio-economic status)*
**Residential area (%)**	NA	x	x	x	x			x	x
**Residential area with multi-storey buildings (%)**	x	NA	x	x	x			**x**	x
**Commercial area (%)**	x	x	NA	x	x			x	x
**Green space (%)**	x	x	x	NA	x			x	x
**NDVI**	x	x	x	x	NA				x
**Rooms per person**						NA	**x**	x	x
**Living space (m²) per person**						**x**	NA	x	x

Table F. Demographic and socio-economic characteristics across all neighbourhoods (median and IQR) and stratified into three groups based on the cumulative number of SARS-CoV-2 infections up to 31.05.2021 in Essen

	Cumulative SARS-CoV-2 infections/1000 inh.			
	All (n=426)	**<9 (n=138)**	**9-<14.5 (n=143)**	**≥14.5 (n=145)**
Demographic	Median [Q1;Q3]			
Number of children below age 6/1000 inh.	*54.3 [46.6;64.0]*	50.8 [43.7;62.2]	52.8 [45.7;62.2]	61.4 [52.0;76.1]
Number of children and adolescents between age 6 and 19/1000 inh.	*116.3 [98.6;139.2]*	104.5 [93.0;123.6]	114.3 [98.1;134.9]	130.8 [108.5;166.5]
Number of elderly above age 70/1000 inh.	*154.9 [113.0;200.4]*	180.4 [150.2;225.8]	148.5 [115.3;192.0]	128.1 [90.4;179.1]
Average age	*44.6 [40.5;48.0]*	47.1 [44.2;49.4]	44.3 [40.9;47.1]	41.9 [38.2;45.6]
Population density (inh./km²)	*5534.8 [3064.2;9134.2]*	4294.1 [2039.7;6390.0]	5987.0 [3077.9;10850.0]	6627.3 [3725.8;10984.6]
Proportion of households with children (%)	*17.7 [14.7;21.2]*	16.7 [14.4;18.9]	16.5 [14.1;19.8]	20.0 [15.8;24.1]
Average household size	*1.9 [1.8;2.1]*	1.9 [1.8;2.0]	1.9 [1.7;2.0]	2.0 [1.8;2.2]
Socio-economic	Median [Q1;Q3]			
Number of persons without German nationality/1000 inh.	*130.0 [65.9;238.8]*	71.8 [44.9;117.4]	139.1 [75.0;219.9]	214.2 [131.2;317.4]
Number of welfare recipients/1000 inh.	*145.9 [55.8;252.8]*	60.3 [27.3;126.0]	136.2 [61.8;243.1]	240.1 [159.7;360.5]
Number of unemployed persons/1000 inh.	*42.7 [19.5;70.2]*	20.4 [13.7;39.8]	42.1 [22.2;67.4]	65.5 [43.4;91.2]

Table G. Crude associations between demographic and socio-economic variables and cumulative SARS-CoV-2 infections in neighbourhoods in Essen (n=426) estimated via ordinary least squares (OLS) regression models and Moran's I of the regression residuals

	β	95% CI	Std. Err.	Adj. R^2	Moran's I (p-value)
Demographic					
Number of children below age 6/1000 inh.	0.13	0.09;0.17	0.02	0.07	0.10 (<.001)
Number of children and adolescents between age 6 and 19/1000 inh.	0.07	0.05;0.09	0.01	0.11	0.10 (<.001)
Number of elderly above age 70/1000 inh.	-0.02	-0.04;-0.01)	0.005	0.03	0.10 (<.001)
Average age	-0.48	-0.63;-0.34)	0.07	0.09	0.08 (<.01)
Population density (inh./km²)	0.0002	0.00008;0.0004	0.00007	0.02	0.16 (<.001)
Proportion of households with children (%)	0.40	0.27;0.53	0.07	0.08	0.14 (<.001)
Average household size	9.20	6.41;12.0	1.42	0.09	0.19 (<.001)
Socio-economic					
Number of persons without German nationality/1000 inh.	0.02	0.02;0.03	0.003	0.15	0.07 (<.01)
Number of welfare recipients/1000 inh.	0.02	0.02;0.03	0.002	0.19	0.01 (0.26)
Number of unemployed persons/1000 inh.	0.10	0.08;0.12	0.01	0.17	0.02 (0.20)

Table H. Crude associations between demographic and socio-economic variables and cumulative SARS-CoV-2 infections in neighbourhoods in Essen (n=426) estimated via mixed spatial lag regression models (SLM)

	β	95% CI	Std. Err.	Pseudo R^2
Demographic				
Number of children below age 6/1000 inh.	0.08	0.03;0.13	0.02	0.13
Number of children and adolescents between age 6 and 19/1000 inh.	0.06	0.03;0.08	0.01	0.14
Number of elderly above age 70/1000 inh.	0.004	-0.01;0.02	0.007	0.12
Average age	-0.24	-0.43;-0.05)	0.10	0.13
Population density (inh./km²)	0.0002	-0.00001;0.0004	0.0001	0.07
Proportion of households with children (%)	0.37	0.21;0.52	0.08	0.12
Average household size	11.79	8.63;14.95	1.61	0.17
Socio-economic				
Number of persons without German nationality/1000 inh.	0.02	0.01;0.03	0.004	0.16
Number of welfare recipients/1000 inh.	0.02	0.01;0.03	0.003	0.20
Number of unemployed persons/1000 inh.	0.07	0.04;0.10	0.02	0.18

Table I. Crude associations between green space components and cumulative SARS-CoV-2 infections in neighbourhoods in Essen (n=426) estimated via mixed spatial lag regression models (SLM)

	β	95% CI	Std. Err.	Pseudo R^2
Public and private green spaces and parks (%)	0.08	0.00;0.16	0.04	0.10
Open spaces near houses (%)	-0.18	-0.31;-0.05	0.07	0.09
Meadows and pastures (%)	0.006	-0.20;0.21	0.10	0.09
Forest (%)	-0.05	-0.16;0.05	0.05	0.09
